# Pre-transplant Transcriptional Signature in Peripheral Blood Mononuclear Cells of Acute Renal Allograft Rejection

**DOI:** 10.3389/fmed.2021.799051

**Published:** 2022-01-07

**Authors:** Wenyu Xiang, Shuai Han, Cuili Wang, Hongjun Chen, Lingling Shen, Tingting Zhu, Kai Wang, Wenjie Wei, Jing Qin, Nelli Shushakova, Song Rong, Hermann Haller, Hong Jiang, Jianghua Chen

**Affiliations:** ^1^Kidney Disease Center, College of Medicine, The First Affiliated Hospital, Zhejiang University, Hangzhou, China; ^2^Key Laboratory of Nephropathy, Hangzhou, China; ^3^Institute of Nephropathy, Zhejiang University, Hangzhou, China; ^4^Zhejiang Clinical Research Center of Kidney and Urinary System Disease, Hangzhou, China; ^5^School of Pharmaceutical Science, Sun Yat-sen University, Shenzhen, China; ^6^Department of Nephropathy, School of Medicine, Shanghai Ruijin Hospital, Shanghai Jiaotong University, Shanghai, China; ^7^Department of Nephrology, Hannover Medical School, Hannover, Germany

**Keywords:** actue renal allograft rejection, RNA-Seq, bioinformatics, biomarker, PBMCs

## Abstract

Acute rejection (AR) is closely associated with renal allograft dysfunction. Here, we utilised RNA sequencing (RNA-Seq) and bioinformatic methods to characterise the peripheral blood mononuclear cells (PBMCs) of patients with acute renal allograft rejection. Pretransplant blood samples were collected from 32 kidney allograft donors and 42 corresponding recipients with biopsies classified as T cell-mediated rejection (TCMR, *n* = 18), antibody-mediated rejection (ABMR, *n* = 5), and normal/non-specific changes (non-AR, *n* = 19). The patients with TCMR and ABMR were assigned to the AR group, and the patients with normal/non-specific changes (*n* = 19) were assigned to the non-AR group. We analysed RNA-Seq data for identifying differentially expressed genes (DEGs), and then gene ontology (GO) analysis, Reactome, and ingenuity pathway analysis (IPA), protein—protein interaction (PPI) network, and cell-type enrichment analysis were utilised for bioinformatics analysis. We identified DEGs in the PBMCs of the non-AR group when compared with the AR, ABMR, and TCMR groups. Pathway and GO analysis showed significant inflammatory responses, complement activation, interleukin-10 (IL-10) signalling pathways, classical antibody-mediated complement activation pathways, etc., which were significantly enriched in the DEGs. PPI analysis showed that IL-10, VEGFA, CXCL8, MMP9, and several histone-related genes were the hub genes with the highest degree scores. Moreover, IPA analysis showed that several proinflammatory pathways were upregulated, whereas antiinflammatory pathways were downregulated. The combination of NFSF14+TANK+ANKRD 33 B +HSPA1B was able to discriminate between AR and non-AR with an AUC of 92.3% (95% CI 82.8–100). Characterisation of PBMCs by RNA-Seq and bioinformatics analysis demonstrated gene signatures and biological pathways associated with AR. Our study may provide the foundation for the discovery of biomarkers and an in-depth understanding of acute renal allograft rejection.

## Introduction

Kidney transplantation is the optimal choice for patients with end-stage renal disease (ESRD). However, acute rejection (AR) cannot be avoided easily when transplanting tissue or cells from a genetically different donor to the graft recipient because the alloantigen of the donor induces an immune response against the graft in the recipient (1).

Acute rejection can occur at any time following transplantation, usually within days to weeks. It is classified as antibody-mediated rejection (ABMR) or acute T cell-mediated rejection (TCMR). In recent years, the overall incidence of AR has decreased, and graft survival has improved with the use of effective immunosuppressive therapy. Currently, AR occurs in approximately 10–20% of cases; however, significant improvement in long-term allograft survival rates remains unrealised (2–4). It has been reported that graft failures occur if AR occurs, even after immunosuppressive treatment, and each episode of rejection is closely associated with a poor graft survival rate (5, 6).

Therefore, investigation into the signature of AR recipients is crucial for understanding the potential pathogenesis and identifying effective biomarkers of rejection. Recently, there have been many studies on biomarkers of allograft rejection. For example, many transplant centres apply donor-derived free DNA for testing AR, which can be positive even before the actual rise in serum creatinine. Some biomarkers detected by the peripheral blood mRNA assay and proteomics methods also showed good accuracy and sensitivity in the diagnosis of various types of rejection (7–9).

RNA sequencing (RNA-Seq) is a molecular tool widely utilised by researchers to analyse global transcriptional changes, deduce pathogenic mechanisms, and discover biomarkers (10). Bioinformatics analysis provides multiperspective methods in data mining, including gene ontology (GO) and pathway analysis, protein–protein interaction (PPI) networks, and some other methods.

In this study, we were able to identify the transcriptional signature in peripheral blood mononuclear cells (PBMCs) of recipients with AR, which helped to distinguish these cells from those of non-AR recipients. Through bioinformatics analysis, several genes and pathways were found to be significantly different between AR and non-AR recipients, including interleukin-10 (IL-10), VEGFA, CXCL8, and histone-related genes, and also IL-10 signalling pathways. Moreover, we found a combination of four genes which could be used to accurately diagnose AR. Thus, this study may provide the basis for further investigations into allograft rejection.

## Materials and Methods

### Study Design, Patient Population, and Sample Collection

Forty-two patients with ESRD who underwent kidney transplantation at the Kidney Disease Centre of the First Affiliated Hospital of Zhejiang University from 01 January 2018 to 31 January 2019 were selected. Inclusion criteria were patients who were (1) ≥18 years of age, regardless of gender and ethnicity; (2) with ESRD undergoing treatment for ≥3 months; and (3) voluntarily joined the study and signed informed consent. Exclusion criteria were patients with (1) acute kidney injury, (2) active inflammatory diseases, (3) other concomitant diseases (such as malignant tumours), and (4) pregnant and lactating women. Based on these criteria, 32 donors also participated in the RNA-Seq cohort study. PBMCs were isolated from the blood of 32 donors and 42 recipients, including 23 biopsy-proven AR recipients (TCMR, *n* = 18; ABMR, *n* = 5) and 19 non-AR recipients with stable kidney function and a normal histology. The classifications for the histopathological diagnosis of renal allograft biopsy were based on the Banff 2017 classification (11). Approximately 3–5-ml peripheral blood samples were stored at −80°C until further use, and kidney allograft biopsies were performed with the help of ultrasound. This study was approved by the Institutional Review Board of the Zhejiang University School of Medicine. The patients or participants provided written informed consent to participate in the study.

### RNA-Seq Experiments and Data Analysis

Total RNA (1000 ng) was extracted from PBMCs using TRIzol reagent (Invitrogen) according to the manufacturer's protocol. The quantity and quality of the RNA isolated from the PBMCs were measured using a NanoDrop2000 spectrophotometer (Thermo Fisher Scientific, Waltham, MA). The Agilent 2100 Bioanalyzer (Agilent Technologies Inc., Santa Clara, CA, USA) was used to measure RNA integrity, reported as the RNA integrity number.

MicroRNA-Seq was performed on an Illumina Hiseq X Ten sequencer by following the manufacturer's protocol (Illumina Inc.). The raw RNA-Seq data were processed as follows: clean reads of good quality were first aligned to human reference databases, namely the hg38 human genome, exon, splicing junction segment splicing junction, and contamination databases including ribosomal and mitochondrial RNA sequences using the BWA alignment algorithm. The feature count was used to count the read numbers mapped to each gene. The read counts were log_2_-transformed, quantile-normalised, and corrected for experimental batch effects using the ComBat R package to compare transcription levels across samples. Then, the normalised bulk RNA-Seq expression data (FPKM) of each gene were calculated based on the length of the gene and read count mapped to said gene. Differential gene expression analysis between patients with AR and non-AR was performed using the Limma package in R (12). A log_2_ fold change (FC) of 1 and *p*-value of 0.05 were set as the threshold for significantly differentially expressed genes (DEGs).

### Gene Ontology Analysis and Pathway Enrichment Analysis of the DEGs

Gene ontology analysis was applied to analyse the main function of the DEGs using DAVID tools (https://david.ncifcrf.gov/home.jsp), which provides a comprehensive set of functional annotation tools for investigators to understand the biological meaning behind a large list of genes (13, 14). Pathway enrichment analysis was performed using Reactome (https://reactome.org/) which is a free, open-source, curated, and peer-reviewed pathway database (15). The significant pathways and GO items, including biological process (BP), cellular component (CC), and molecular function (MF) were defined as pathways with *p* < 0.05.

### PPI Network of the DEGs

To identify the hub genes and examine the interactions between the DEGs, a PPI network was generated using STRING software (https://string-db.org/). For the search parameters, the organism queried was set to *homo sapiens*, the required confidence score was set to 0.9 (highest confidence), and the interactors shown were set to no more than 20. The edges reflected the strength of evidence and were drawn with up to three different thickness values: medium (0.400), high (0.700), and highest (0.900). The false discovery rate was set to 0.05. The search results were then imported into Cytoscape (version 3.8.2) for further analysis. The hub genes were identified using the CytoHubba plugin with the degree score, and the top 10 genes were finally selected (16).

### Cell-Type Enrichment Analysis Using XCell and CIBERSORT

Cell-type abundance estimation of the RNA-Seq data was determined using xCell (https://xcell.ucsf.edu/) (17), a bioinformatics tool that generates cell-type enrichment scores (ESs) based on gene expression data for 64 immune and stromal cell types, using the FPKM as the input. Relative cell-type abundance was quantified and visualised for all samples. The abundance of each cell type between non-AR and AR was compared using the Wilcoxon rank-sum test. Cell types with a *p* < 0.1 were considered to be significantly differentially enriched.

The deconvolution method using the Cell-type Identification By Estimating Relative Subsets of known RNA Transcripts (CIBERSORT) algorithm was also performed to estimate the population percentage of immune cells for each sample from the bulk RNA-Seq profile (https://cibersort.stanford.edu/) (18). Based on the assumption that the expression value for each immune cell marker in the bulk RNA-Seq is the weighted sum of each cell type in the expression base matrix of 547 immune cell markers in 22 sorted pure immune cells (547 × 22 matrix), CIBERSORT was used to perform a support vector regression (SVR) on the bulk expression value of marker genes to calculate the weight of each cell type, which was then converted into cell population percentages. A Student's *t*-test was used to determine the population change for each cell type between patients with AR and non-AR at the cut-off of *p* < 0.05.

### Ingenuity Pathway Analysis (IPA)

We utilised an ingenuity pathway analysis (IPA) to further mine the potential pathways related to the DEGs. The IPA is a web-based software application for the analysis, integration, and interpretation of data derived from gene expression experiments, including RNA-Seq, microRNA and SNP microarrays, metabolomics, proteomics, and small-scale experiments that generate gene and chemical lists. A downstream effect analysis was used to predict cellular functions, disease processes, and other phenotypes impacted by patterns in the analysed data set. In addition, an upstream regulator analysis was done to identify regulators (transcription factors, cytokines, kinases, etc.) directly linked to the targets in the analysed data and whose activation or inhibition may account for the observed changes. A positive Z-score indicated that the pathway was promoted, whereas a negative value indicated that the pathway was suppressed.

### Statistical Analyses

Continuous variables with normal distribution were expressed as mean ± SD, and variables with a skewed distribution were represented by the median (interquartile range). Categorical variables were expressed in terms of rate (%) or composition ratio (%). The comparison between the two groups of continuous variables was analysed using a Student's *t*-test, and the comparison between the categorical variables was performed using the chi-squared test. When the two-sided test yielded a *p* < 0.05, the difference was considered statistically significant. All data were statistically analysed using SPSS Statistics v20 (IBM Analytics), and statistical charts were created using GraphPad Prism software (version 8.0.1; GraphPad Software, San Diego, CA) or R software (v. 3.3.2).

## Results

### Characteristics of the PBMCs RNA-Seq Cohort

We performed RNA-Seq on 74 pretransplant blood PBMC samples collected from 32 kidney allograft donors and 42 corresponding recipients, whose kidney biopsies were classified as AR, including 18 and 5 patients with TCMR and ABMR, respectively, and also 19 patients with non-AR. The demographic and clinical characteristics of the patients are presented in Table 1. The dialysis vintage was significantly longer (*p* = 0.042), and the glomerular filtration rate (GFR) was significantly reduced (*p* = 0.019) in AR recipients than in non-AR recipients. There were no statistically significant differences between the two groups in terms of recipient or donor age or sex, induction type, kidney disease, HLA mismatch, cold ischaemia time, blood urea nitrogen, serum creatinine, urine protein, and uric acid (*p* > 0.05).

**Table 1:** Demographic and clinical characteristics of kidney allograft recipients.

**Characteristics**	**Total**	**Non-AR (*n* = 19)**	**AR (*n* = 23)**	***p*-value**
Recipient age (years)	38.2 ± 1.7	38.3 ± 9.2	38.5 ± 11.3	0.067
Recipient sex (male %)	61.9	63.2	60.9	0.879
Dialysis vintage (months)	11.4 (2.75–51.95)	7.45 (0–23.25)	32.95 (5.83–66.5)	**0.042**
**Induction type**, ***n*** **(%)**
Antithymocyte globulin	12 (28.57)	4 (21.05)	8 (34.78)	
Basiliximab	29 (69.05)	15 (78.95)	14 (60.87)	0.301
Both	1 (2.38)	0 (0)	1 (4.35)	
**Kidney disease**, ***n*** **(%)**
Glomerulonephritis	31 (73.81)	16 (84.21)	15 (65.22)	
Hypertension	4 (9.52)	0 (0)	4 (17.39)	0.086
Polycystic kidney disease	1 (2.38)	0 (0)	1 (4.35)	
Others	6 (14.29)	3 (15.79)	3 (13.04)	
Donor age (years)	53 (41.75–58)	55 (46.5–59)	49 (38.25–57.5)	0.187
Donor sex (male %)	57.1	52.6	60.9	0.591
**Deceased donor (Y/N)**, ***n*** **(%)**
Y	16 (38.1)	3 (15.79)	13 (56.52)	**0.017**
N	26 (61.9)	16 (84.21)	10 (43.48)	
**HLA overall mismatch**, ***n*** **(%)**
Mismatch (0)	0 (0)	0 (0)	0 (0)	
Mismatch (l−2)	15 (35.71)	7 (36.84)	8 (34.78)	0.385
Mismatch (3–4)	24 (57.14)	12 (63.16)	12 (52.17)	
**HLA-A mismatch**, ***n*** **(%)**
Mismatch (0)	9 (21.43)	5 (26.32)	4 (17.39)	0.270
Mismatch (l)	31 (73.81)	14 (73.68)	17 (73.91)	
Mismatch (2)	2 (4.76)	0 (0)	2 (8.7)	
**HLA-B mismatch**, ***n*** **(%)**
Mismatch (0)	6 (14.29)	2 (10.53)	4 (17.39)	
Mismatch (l)	30 (71.43)	16 (84.21)	14 (60.87)	0.567
Mismatch (2)	6 (14.29)	1 (5.26)	5 (21.74)	
**HLA-DR mismatch**, ***n*** **(%)**
Mismatch (0)	3 (7.14)	2 (10.53)	1 (4.35)	0.622
Mismatch (l)	32 (76.19)	14 (73.68)	18 (78.26)	
Mismatch (2)	7 (16.7)	3 (15.79)	4 (17.39)	
CIT (mins)	180 (120–480)	150 (120–255)	275 (120–585)	0.065
**DGF(Y/N)**, ***n*** **(%)**
Y	1 (2.38)	0 (0)	1(4–35)	1.000
N	41 (97.62)	19 (100)	22 (95.65)	
BUN (mmol/L)	18.33 ± 0.98	16.32 ± 5.57	19.76 ± 6.64	0.751
SCR (umol/L)	775 ± 47	701 ± 297	820 ± 263	0.523
GFR (mL/min/1.73 m2)	7.45(5.13–10.45)	9.7 (6.5–12.43)	5.85 (4.68–8.35)	**0.019**
UPRO (g/L)	2.54 ± 0.23	2.39 ± 1.01	2.49 ± 1.66	0.050
UA (umol/L)	369 ± 17	352 ± 102	382 ± 112	0.866

*Numbers are presented as mean ± SD, median (25–75 percentiles) or count (percentage %). AR, acute rejection; non-AR, non-acute rejection; CIT, cold ischaemia time; DGF, delayed graft function; BUN, blood urea nitrogen; SCR, serum creatinine; GFR, glomerular filtration rate; UPRO, urine protein; UA, uric acid. The bold values represent there are statistical differences between the patients with AR and non-AR*.

### RNA-Seq and Differential Gene Expression Analysis

**Figure 1:**
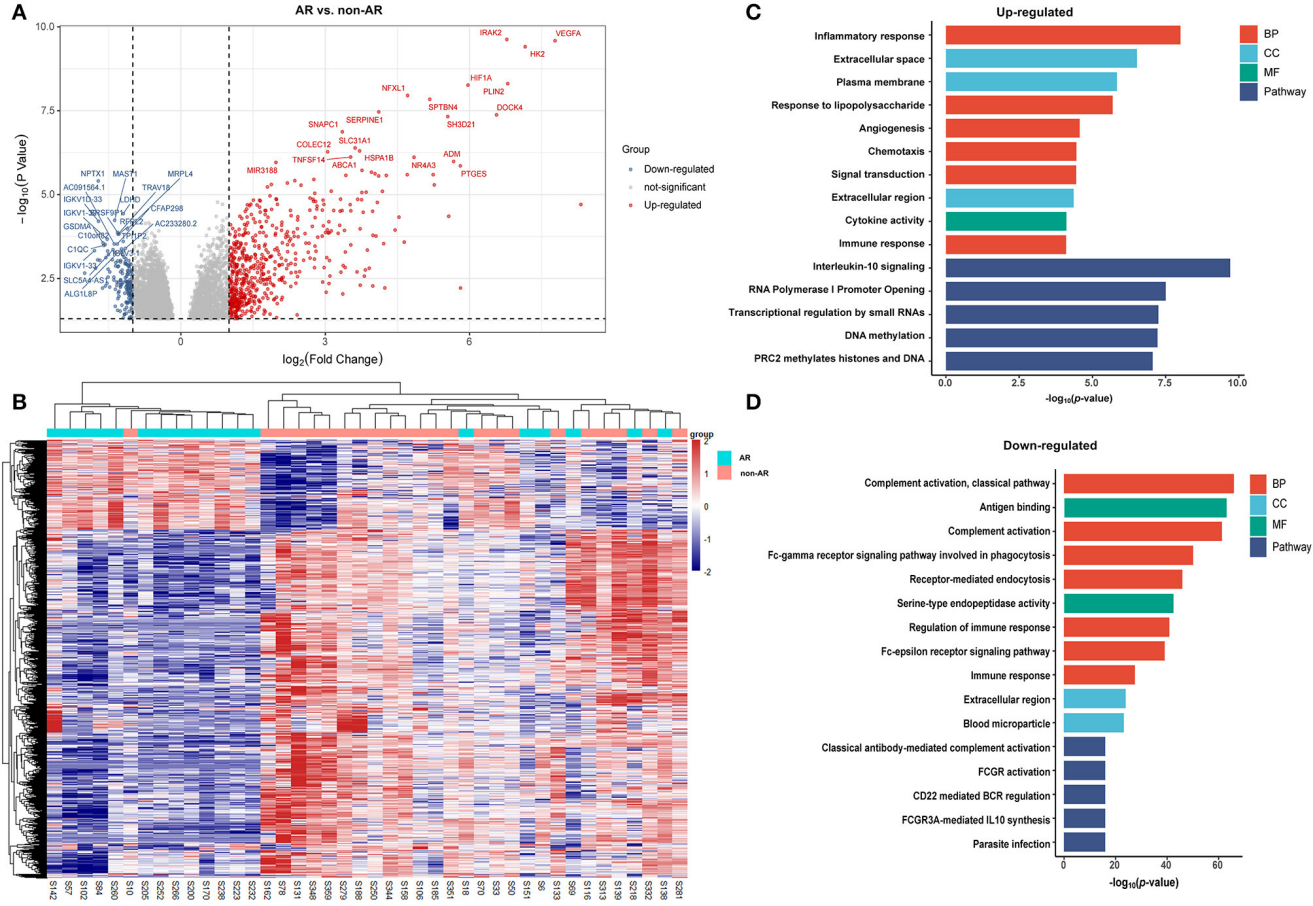
DEGs and GO analysis (AR and non-AR recipients). **(A)** Volcano map of DEGs in AR vs. non-AR recipients. Red represents upregulated genes, and blue represents downregulated genes. A total of 975 DEGs were identified, among which 776 were upregulated and 199 were downregulated in patients with AR compared with patients with non-AR. **(B)** Hierarchical clustering heatmap analysis of DEGs. Red represents upregulated genes, and blue represents downregulated genes. **(C)** GO and pathway analysis of upregulated DEGs. GOs of inflammatory response and IL-10 signalling were the most significant items. **(D)** GO and pathway analysis of downregulated DEGs. GOs of the complement activation and classical antibody-mediated complement activation pathway were the most significant items.

We identified 975 genes as those differentially distinguishing patients with AR from patients with non-AR using log_2_ FC > 1 and *p* < 0.05, as the thresholds for differential gene expression (Supplementary Table). Among these, 776 were upregulated and 199 were downregulated in patients with AR compared to patients with non-AR. The volcano plots showed differences in PBMC gene expression between AR and non-AR (Figure 1A), and only the top 20 significantly expressed genes are shown in Figure, including several important genes, such as C1QC, VEGFA, IRAK2, HIF1A, and SERPINE1, which are closely associated with the immune system, growth of peripheral blood vessels, suppression of oxidative phosphorylation and fatty acid oxidation, and systemic insulin resistance (19–22).

**Figure 2:**
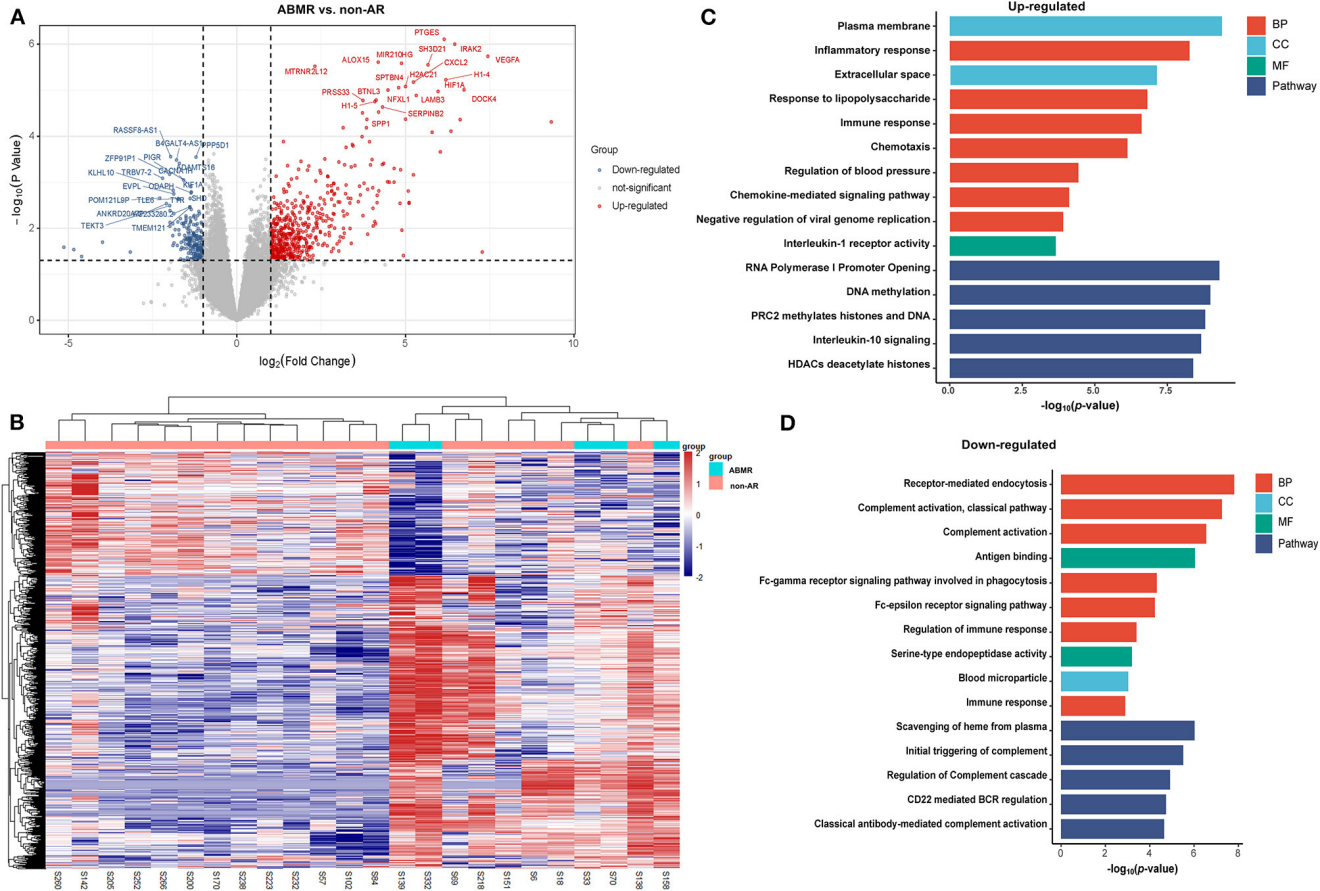
DEGs and GO analysis (ABMR and non-AR recipients). **(A)** Volcano map of DEGs in ABMR vs. non-AR recipients. Red and blue represent upregulated and downregulated genes, respectively. A total of 1,036 genes were identified as DEGs in PBMCs of patients with ABMR, among which 730 were upregulated and 306 were downregulated. **(B)** Hierarchical clustering heatmap analysis of DEGs. Red represents upregulated genes, and blue represents downregulated genes. **(C)** GO and pathway analysis of upregulated DEGs. GOs of inflammatory response and IL-10 signalling were significantly enriched. **(D)** GO and pathway analysis of downregulated DEGs. GOs of the complement activation and classical antibody-mediated complement activation pathway were significantly enriched.

**Figure 3:**
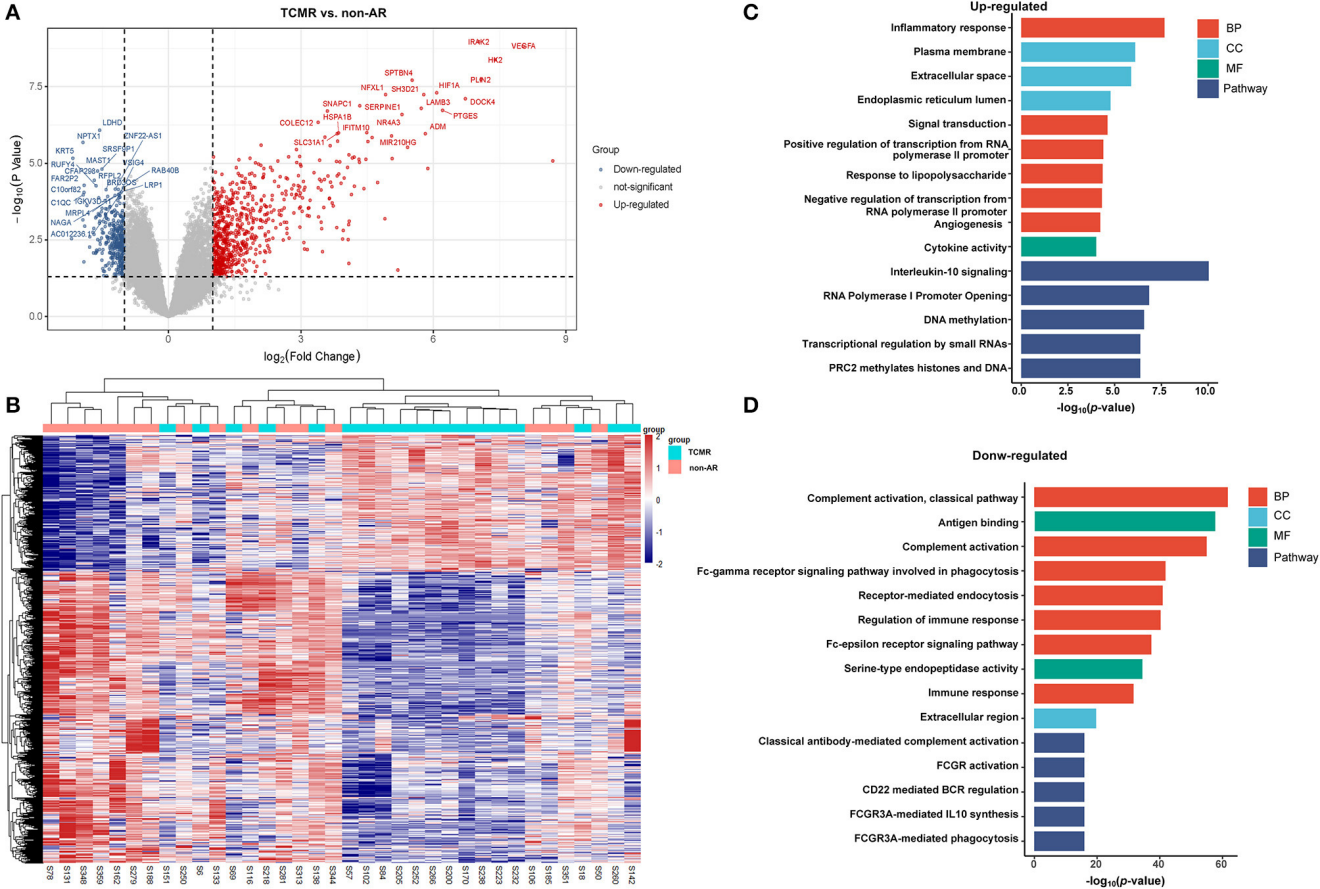
DEGs and GO analysis in (TCMR and non-AR recipients). **(A)** Volcano map of DEGs in TCMR vs. non-AR recipients. Red represents upregulated genes, and blue represents downregulated genes. A total of 1,375 genes were identified as DEGs in PBMCs of patients with TCMR, among which 936 were upregulated and 439 were downregulated. **(B)** Hierarchical clustering heatmap analysis of DEGs. Red represents upregulated genes, and blue represents downregulated genes. **(C)** GO and pathway analysis of upregulated DEGs. GOs of inflammatory response and IL-10 signalling were the most significant enrichments. **(D)** GO and pathway analysis of downregulated DEGs. GOs of the complement activation and the classical antibody-mediated complement activation pathway were the most significant enrichments.

**Figure 4:**
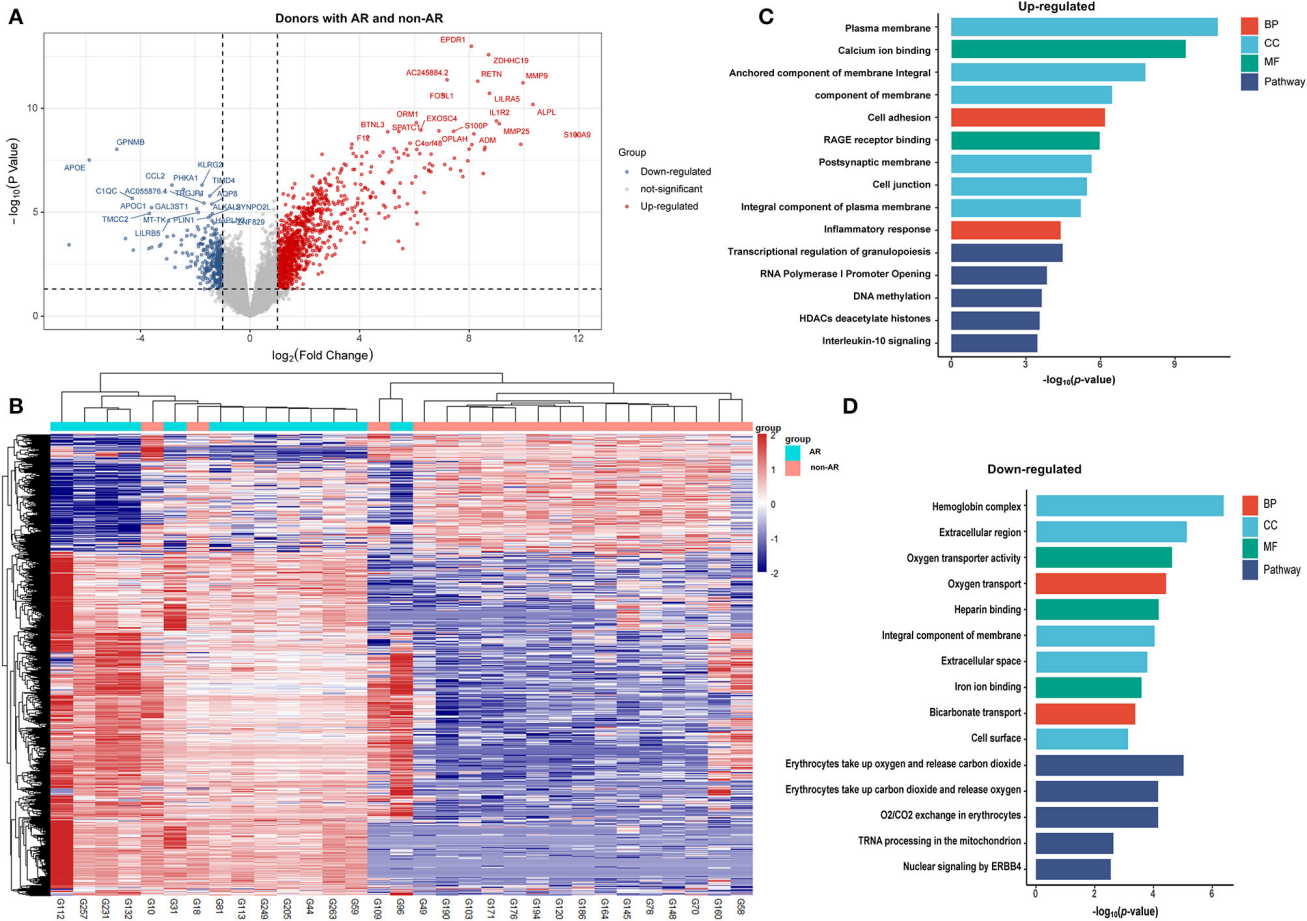
DEGs and GO analysis (donors with AR and non-AR). Volcano map of DEGs in TCMR vs. non-AR recipients. Red represents upregulated genes, and blue represents downregulated genes. A total of 1472 upregulated and 505 downregulated genes were identified as DEGs in donors with AR (*n* = 13) compared with non-AR (*n* = 18). **(B)** Hierarchical clustering heatmap analysis of DEGs. Red represents upregulated genes, and blue represents downregulated genes. **(C)** GO and pathway analysis of upregulated DEGs. **(D)** GO and pathway analysis of downregulated DEGs.

A total of 1,036 genes were identified as DEGs distinguishing the PBMCs of patients with ABMR from those of non-AR individuals, among which 730 were upregulated and 306 were downregulated (Figure 2A). Several DEGs detected in the AR group were also significantly expressed in the PBMCs of patients with ABMR, including EGFA, IRAK2, and HIF1A. To distinguish patients with TCMR from the non-AR group, a total of 1,375 genes were identified as DEGs, among which 936 were upregulated and 439 were downregulated (Figure 3). Of note, the top 20 DEGs identified here were highly similar to those for the AR group, suggesting that TCMR may play a more important role in the process of AR than ABMR (Figure 3A). As shown in Figure 4A, a total of 1,472 upregulated and 505 downregulated genes were identified as DEGs distinguishing AR (*n* = 13) from patients with non-AR (*n* = 18). Hierarchical clustering heatmap analyses of DEGs among different groups were shown in Figures 1B, 2B, 3B, 4B.

### Gene Ontology Analysis and Pathway Enrichment Analysis of the DEGs Patients With AR vs. Non-AR

The DEGs were further analysed using DAVID tools, which were also used for the analysis of BPs, CCs, and MF. As shown in Figure 1C, GO analysis of upregulated DEGs showed several important BPs and MFs, including cytokine activity, inflammatory responses, responses to lipopolysaccharide, angiogenesis, chemotaxis, signal transduction, and immune responses. In contrast, significant GO downregulation in AR included the processes of complement activation, antigen binding, and the Fc-gamma receptor signalling pathway involved in phagocytosis and regulation of the immune response. Among these, GOs associated with immunity were significantly enriched in both upregulated and downregulated genes, suggesting that the dysfunction of the immune system plays an important role in the process of AR. Pathway enrichment analysis of the DEGs in the AR group compared with the non-AR group was performed using the Reactome pathway analysis. As shown in Figure 1C, the IL-10 signalling pathway was significantly enriched in the upregulated genes. However, classical antibody-mediated complement activation, FCGR3A-mediated IL-10 synthesis, FCGR activation, and CD22-mediated BCR regulation pathways were significantly enriched in the downregulated genes (Figure 1D). Among these were classical antibody-mediated complement activation pathways previously identified for AR (23). The remaining GO and pathway enrichments are described in the Supplementary Materials. Notably, IL-10-related pathways were significantly enriched in both upregulated and downregulated pathways, suggesting that IL-10 and IL-10 signalling pathways may contribute to the process of AR.

### Patients With ABMR vs. Non-AR

As shown in Figure 2C, the GOs of inflammatory response, response to lipopolysaccharides, immune response, chemotaxis, and chemokine-mediated signalling pathways were significantly enriched in upregulated genes. In contrast, receptor-mediated endocytosis, complement activation (classical pathway), and antigen binding pathways were significantly enriched in downregulated genes (Figure 2D). Pathway analysis showed that the RNA polymerase I promoter opening, DNA methylation, PRC2 methylates histones, and DNA and IL-10 signalling pathways were significantly enriched in upregulated genes. In contrast, scavenging of haeme from plasma, initial triggering of complement, regulation of complement cascade, CD22-mediated BCR regulation, and classical antibody-mediated complement activation pathways were significantly enriched in downregulated genes. The rest of the GO and pathway items are described in the Supplementary Materials.

### Patients With TCMR vs. Non-AR

As shown in Figure 3C, GO items of inflammatory responses, angiogenesis, chemotaxis, and cytokine activity were significantly enriched in upregulated genes. In contrast, GOs of complement activation (classical pathway), antigen binding, complement activation, receptor-mediated endocytosis, regulation of immune response, etc. were significantly enriched in downregulated genes (Figure 3D). Pathway analysis showed that IL-10 signalling, RNA polymerase I promoter opening, DNA methylation, and transcriptional regulation by small RNA pathways were significantly enriched in upregulated genes. In contrast, classical antibody-mediated complement activation, FCGR activation, CD22-mediated BCR regulation, and FCGR3A-mediated IL-10 synthesis pathways were significantly enriched in downregulated genes. The rest of the GO and pathway items are described in the Supplementary Material. These GOs and pathways were very similar to the items identified in the AR group, suggesting that TCMR may play a more important role in the occurrence of AR.

### Donors With AR vs. Non-AR Individuals

As shown in Figure 4C, the GOs of calcium ion binding, anchored component of membrane, cell adhesion, inflammatory response, etc. were significantly enriched in upregulated genes. In contrast, the GOs of oxygen transporter activity, oxygen transport, and heparin binding were significantly enriched in downregulated genes (Figure 4D). Pathway analysis showed that the transcriptional regulation of granulopoiesis, DNA methylation, and IL-10 signalling, among others, were significantly enriched in upregulated genes. Of note, although IL-10 signalling was identified in donors with AR, the degree was far smaller than that in recipients with AR, ABMR, or TCMR. On the other hand, erythrocytes take up oxygen and release carbon dioxide, and tRNA processing in the mitochondria, etc., were significantly enriched in downregulated genes.

### Enrichment of Immune Cell Types in PBMCs

Owing to the fact that the PBMC-RNA-Seq profile is likely a mixture of RNA from multiple cell types, we used bulk RNA cell-type enrichment analysis using the gene expression data to recover the identity of the cell types found in AR and non-AR samples with the gene signature expression-based cell-type enrichment tool xCell, and also CIBERSORT.

**Figure 5:**
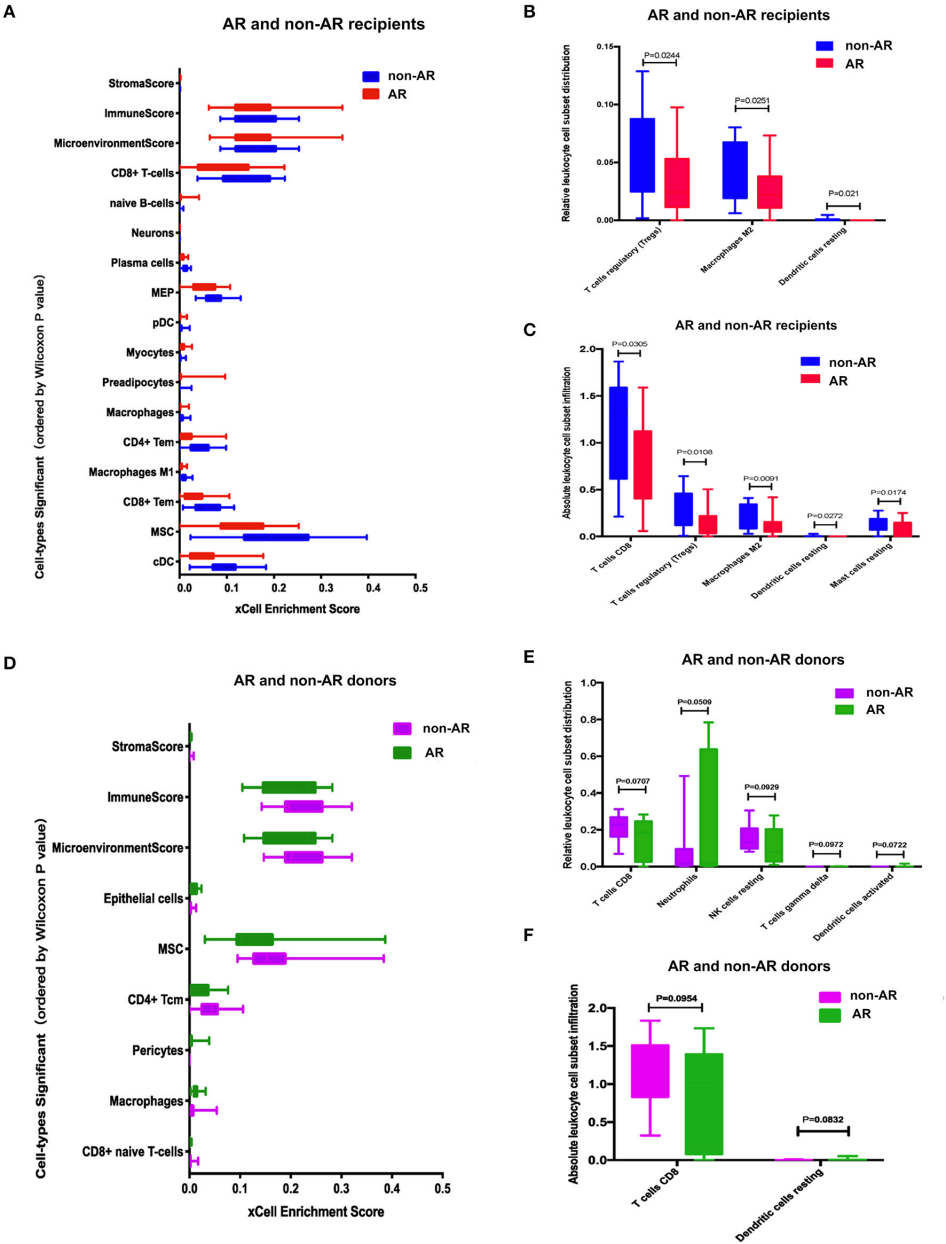
Cell-type enrichment analysis using xCell and CIBERSORT. **(A)** Cell-type enrichment analysis of the recipients RNA-Seq data determined using xCell, a bioinformatics tool that generates cell-type ESs based on gene expression data for 64 immune and stromal cell types. The *x*-axis depicts the xCell ES, and the *y*-axis lists 14 of the 64 cell types that were differentially enriched (FDR < 0.1, Wilcoxon test with Benjamini-Hochberg correction) in AR vs. non-AR recipients. Box plots of the immune score (composite score of immune cell types) and the microenvironment score (composite scores of immune cell types and stromal cell types) are also shown. **(B, C)** Immune cell enrichment analysis of the recipients using CIBERSORT. The bar chart shows the relative **(B)** and absolute **(C)** leukocyte cell subset population differences between AR and non-AR recipients. The population percentages of CD8+ T cells and Tregs were deconvoluted from the RNA-Seq using the expression profiles of sorted immune cells. **(D)** Cell type enrichment analysis using xCell between donors with AR and non-AR. **(E–F)** Immune cell enrichment analysis of the donors using Cibersort. The bar chart shows the relative **(E)** and absolute **(F)** leukocyte cell subset population differences between donors with AR and non-AR.

Cell-type ESs across 64 immune and stromal cell types were obtained for PBMCs using xCell. Our data analysis demonstrated that there were 14 cell types differentially enriched in AR vs. non-AR recipients (FDR < 0.1), with 4 of 14 cell types positively enriched and the remaining 10 negatively enriched (Figure 5A).

The relative or absolute leukocyte cell subset population percentages were deconvoluted from RNA-Seq using the expression profiles of sorted immune cells. Figure 5B shows the relative cell subset distribution difference in Tregs, macrophages M2 cell, and dendritic cells resting populations between AR and non-AR recipients based on gene expression. Figure 5C shows the absolute cell subset infiltration difference in T cells CD8, Tregs, macrophages M2, dendritic cells, and resting mast cells between AR and non-AR recipients based on gene expression.

Cell types were also quantitated following analysis of AR vs. non-AR donors, and six cell types were differentially enriched at FDR < 0.1 (Figure 5D). Figure 5E shows the relative cell subset infiltration difference in T cells CD8, neutrophils, resting NK cells, T cell gamma delta, and dendritic cell-activated populations between AR and non-AR donors based on gene expression. Figure 5F shows the absolute cell subset infiltration difference in T cells, CD8, and dendritic cells resting populations between AR and non-AR donors based on gene expression; however, none of the results showed a statistical difference (*p* < 0.05).

### IPA

**Figure 6:**
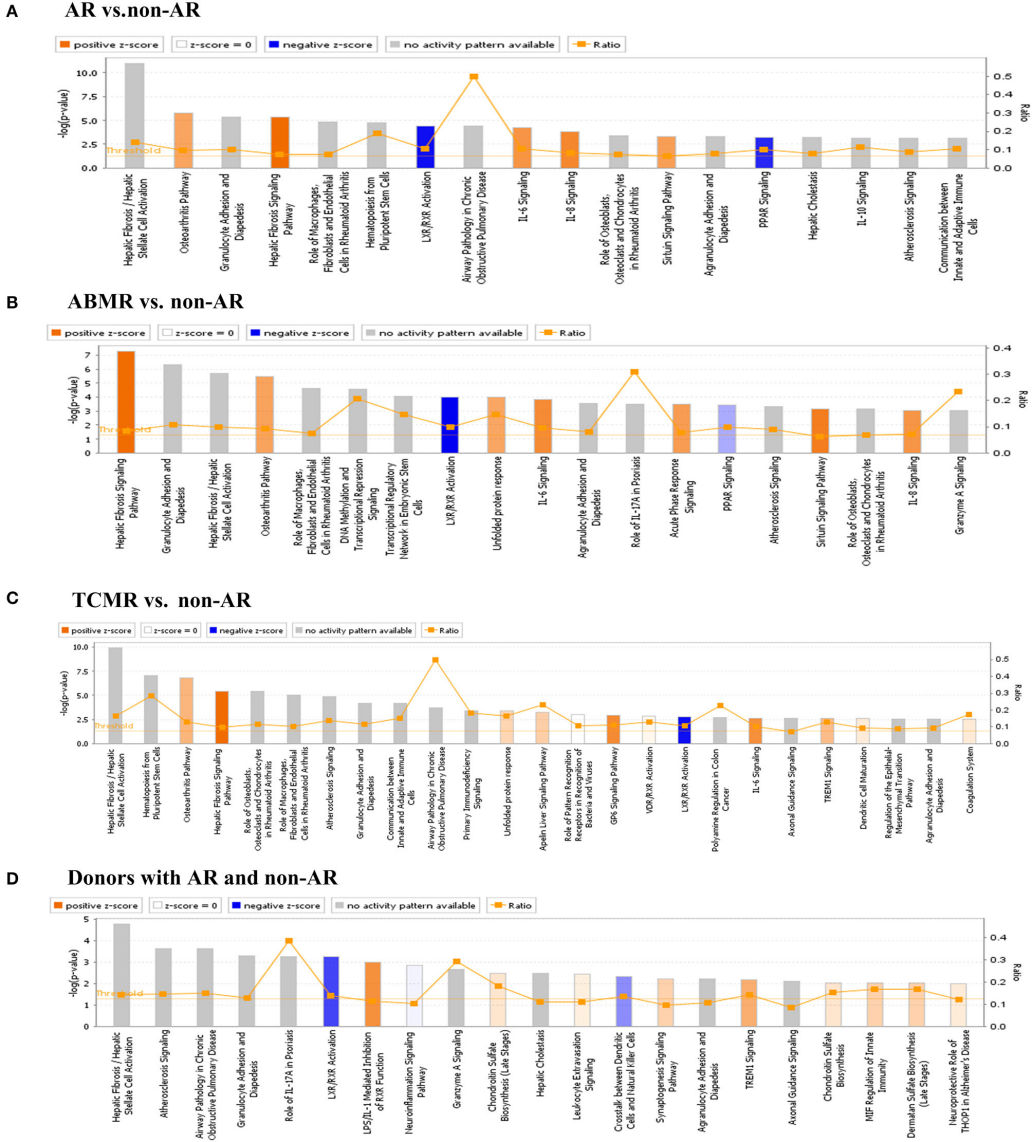
IPA analysis of DEGs. **(A)** IPA analysis of DEGs in AR vs. non-AR group. IL-6 and IL-8 signalling, etc. had a positive Z-score, whereas LXR/RXR activation and PPAR signalling had a negative Z-score. **(B)** IPA analysis of DEGs in ABMR vs. non-AR group. Hepatic fibrosis pathway, IL-6 and IL-8 signalling, etc., had a positive Z-score, whereas LXR/RXR activation and PPAR signalling had a negative Z-score. **(C)** IPA analysis of DEGs in TCMR vs. non-AR group. Hepatic fibrosis signalling pathway and IL-6 signalling, etc. had a positive Z-score, whereas LXR/RXR activation signalling had a negative Z-score. **(D)** IPA analysis of DEGs in donors with AR vs. non-AR group. LPS/IL-1-mediated inhibition of RXR function and TREM1 signalling etc. had a positive Z-score and LXR/RXR activation signalling had a negative Z-score.

Bar charts of enriched canonical pathways of DEGs were plotted using the IPA tool. The *y*-axis represents the –log_10_ *p*-value of enrichment significance of IPA pathways by Fisher's exact test. We found that LXR/RXR activation was detected with a negative Z-score in the four groups (Figure 6). PPAR signalling was detected with a negative Z-score in both AR vs. non-AR and ABMR vs. non-AR groups. In contrast, the hepatic fibrosis signalling pathway was detected with a positive Z-score in the four groups. The IL-6 signalling pathways were detected in the AR groups (including ABMR and TCMR), but not in the donor group with AR.

### Hub Genes From PPI Network and Receiver Operating Characteristic (ROC) Curves for Distinguishing AR From Non-AR Individuals

**Figure 7:**
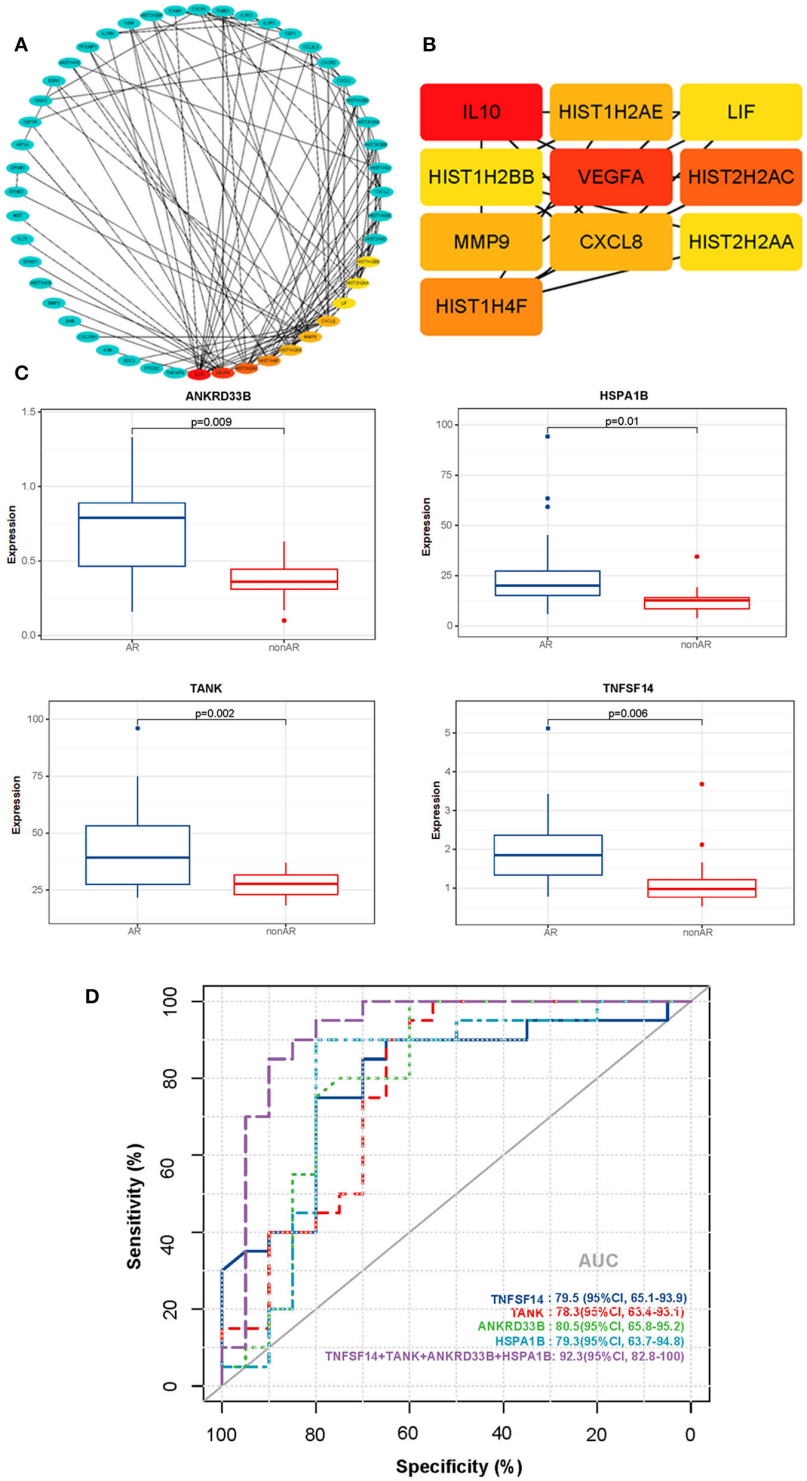
PPI networks of DEGs and ROC curves for diagnosis of AR. **(A)** The PPI network of DEGs detected in AR vs. non-AR groups was performed with Cytoscape. **(B)** The hub genes were identified by the CytoHubba plugin with the top 10-degree score. **(C)** Box plots of the mRNA expression. **(D)** ROC curves were constructed to determine the diagnostic power of DEGs for AR. TNFSF14: AUC 79.5 (95% CI, 65.1–93.9); TANK: AUC 78.3 (95% CI, 63.4–93.1); ANKRD33B: AUC 80.5 (95% CI, 65.8–95.2); HSPA1B: AUC 79.3 (95% CI, 63.7–94.8); NFSF14+TANK+ANKRD33B+HSPA1B: AUC 92.3 (95% CI, 82.8–100).

We utilised STRING and Cytoscape software to further analyse the interaction between the AR and non-AR DEGs. As shown in Figure 7A, several important genes were identified, with high confidence (interaction score >0.9), as part of the predominant network. Subsequently, for identification of hub genes, we performed the PPI network analysis with the CytoHubba plugin using the degree method, and the top 10 genes were identified for further analysis as the hub genes (Figure 7B). Interestingly, several hub genes were members of the histone family including HIST2H2AC, HIST1H4F, HIST1H2AE, HIST2H2AA, and HIST1H2BB, suggesting that it plays an important role in the process of AR.

We also conducted a conventional receiver operating characteristic (ROC) curve analysis to determine the power of DEGs for the diagnosis of AR (Figures 7C,D). Interestingly, four genes (TNFSF14, TANK, ANKRD33B, and HSPA1B) showed good performance in distinguishing between AR and non-AR groups. Moreover, the 4-gene combination reached an ROC AUC of 92.3% (95% CI 82.8–100) with respect to discrimination between AR and non-AR, suggesting its potential clinical usage in monitoring transplant patients.

## Discussion

With the effective use of immunosuppressive drugs, the rate of acute renal graft rejection has declined in recent years. Kidney allograft rejection is associated with molecular changes in renal allograft tissue, which reflect transcription changes in resident cells or changes in cell populations, such as effector T cells, macrophages, and natural killer (NK) cells (24, 25). Therefore, here we investigated the signature in PBMCs of recipients with AR using RNA-Seq and bioinformatics analysis to further explore the potential pathogenesis, identify biomarkers, and provide a basis for follow-up investigations.

In our study, the PPI network showed several significant genes with high confidence, including IL-10, VEGFA, CXCL8, MMP9, and histone-related genes (Figures 7A,B). Chemokines play an important role in coordinating the immune system *via* many BPs, including regulating the migration of immature lymphoid progenitor cells, the recirculation of mature naïve T and B lymphocytes, and the homing of antigen-specific effector T cells. It also regulates the migration of antigen-presenting cells, such as dendritic cells and monocytes or macrophages. Recently, many studies have reported that several members of the CXC family of chemokines show significant differences with different types of allograft rejection. A study revealed that mRNAs for four chemokines (CCL5, CXCL9, CXCL10, and CXCL11) were positively enriched in TCMR urine compared with non-rejection urine. Similarly, mRNAs for six chemokines (CCL2, CCL5, CXCL5, CXCL9, CXCL10, and CCL18) were positively enriched in ABMR urine compared with non-rejection urine (10). Jasper et al. identified and validated a novel eight-gene expression assay (CXCL10, FCGR1A, FCGR1B, GBP1, GBP4, IL15, KLRC1, and TIMP1) that could be used as a non-invasive and effective diagnostic biomarker for ABMR (ROC AUC = 79.9%; 95% CI 72.6–87.2, *p* < 0.0001) (8). Mueller et al. reported that the expression of CXCL10 and CXCL9 was significantly increased in kidney biopsy specimens with TCMR, which was supported by clinical data from multicentre studies of increased urinary CXCL9 and CXCL10 mRNA and protein levels as diagnostic biomarkers of TCMR (26, 27). Additionally, Chen et al. reported that CXCL13 could help to identify AR from a stable group following kidney transplantation. Their study showed that CXCL13 mRNA expression was ten times higher in AR, than that in the stable group (*p* < 0.001), and was hence a good diagnostic biomarker (ROC AUC 0.89; 95% CI: 0.81 0.97). Moreover, the serum protein level of CXCL13 detected by ELISA was 2.2 times higher in the acute group than in the stable group (328.4 vs. 147.6 ng/ml, *p* = 0.002). Our study showed that CXCL8 (also known as IL-8) was significantly increased in PBMCs from the AR group. However, few studies have investigated its performance in patients with AR. Given that several members of the chemokine CXC family have previously been shown to be effective at diagnosing allograft rejection, CXCL8 requires further investigation as a biomarker in the future.

Moreover, our PPI network demonstrated that IL-10 showed strong interactions with other genes, suggesting a contribution to the AR process. Similarly, Verma et al. (10) found that the expression of IL-10 was significantly increased in patients with TCMR compared with patients with non-TCMR. IL-10, a cytokine with antiinflammatory properties, plays a central role in infection by limiting the immune response to pathogens, thereby preventing damage to the host. Dysregulation of IL-10 is linked with susceptibility to numerous infectious and autoimmune diseases in humans and mouse models (28). It is expressed by a variety of cell types including macrophages, dendritic cell subsets, B cells, and several T cell subpopulations, including Th2 and T-regulatory cells (Tregs) and NK cells (29). Deficiency of IL-10 or its receptors results in aberrant immune responses that lead to immunopathology and diseases (30, 31). Such imbalance in pathological vs. regulatory immune networks can result in graft vs. host disease (GVHD), which is a limiting complication of allogeneic stem cell transplantation. IL-10 secretion is dynamically modulated by the availability of antigens, costimulatory signals, cytokines, commensal microbes, and their metabolites in the microenvironment. There were some similarities between GVHD and AR, such as pathological processes involving dysregulation of the immune system and dysfunction of immune cells. These results warrant further, future investigation of the role of IL-10 in the AR processes.

Vascular endothelial growth factor (VEGF) is an essential growth factor that participates in various pathophysiological processes, including embryonic development, repair of traumatised tissue, ischaemia, inflammation, and tumour occurrence by promoting angiogenesis. Tambur et al. (21) and Aharinejad et al. (22) found that VEGF expression is correlated with AR and chronic rejection. On a related note, Berberat et al. (32) found that the use of anti-VEGF reagents could effectively inhibit the progression of AR. Several studies have reported that VEGF regulates many AR-related molecules, both *in vivo* and *ex vivo*, including IL-10, mononuclear cell chemokine-1 (MCP-1), IL-8, E-selectin, ICAM-1, and VCAM-1 (33–35). The infusion of macrophages and lymphocytes stimulates angiogenesis, which in turn promotes inflammation (36). The contribution of VEGF to AR occurrence following liver transplantation is mainly due to the recognition of alloantigens of the donor by T lymphocytes, which can induce a series of immune responses thereby negatively affecting liver transplantation (37). Similarly, we found that VEGFA significantly increased in patients with AR; however, the role of VEGFA in AR following kidney transplantation remains unclear and requires further investigation.

As described previously, both GO and pathway analysis showed that genes related to the immune system, complement activation, and inflammatory response were significantly enriched in the AR group. Classical antibody-mediated complement activation mediates many of the downstream effects of antibodies, which are affected by many factors, including antigen density and configuration ratio, antibody abundance, antibody titre and isotype, and complement regulation by the target tissue. The C1r and C1s serine proteases are transactivated and acquire the ability to cleave C4 into C4a and C4b fragments when C1q binds to IgG-or IgM-containing immune complexes (38). Pathway analysis showed that the IL-10 signalling pathway was significantly enriched in the upregulated genes. As described above, the IL-10 signalling pathway plays an important role in the immune response and may mediate the occurrence of AR. However, the classical antibody-mediated complement activation pathway, including C1QB, C1QC, and many immunoglobulin components, was significantly enriched in downregulated genes, suggesting that the dysfunction of the IL-10 signalling pathway may contribute to the process of AR.

Based on the IPA analysis, the PPAR signalling pathways and LXR/RXR activation were predicted to be downregulated, suggesting that they may play an important role in acute renal allograft rejection. Metabolomics, lipidomics, functional metabolic assays, and single-cell analysis of cultured human macrophages revealed that PPARα regulates macrophage glycolysis, citrate metabolism, and mitochondrial membrane sphingolipid metabolism and suppresses its inflammatory properties. Treatment with the PPARα agonist suppressed the development of vein graft lesions, while silencing of PPARα in macrophages promoted vein graft lesion development (39). Thus, PPAR probably acts as a protective factor in the process of AR, and the suppression of PPAR may promote acute renal allograft rejection. LXR/RXR activation has been reported to have antiangiogenic and anti-inflammatory effects (40, 41). The expression and activation of LXRs in human lymphocytes reduce pro-inflammatory signalling, while activation of LXR using synthetic agonists in monocytes promotes anti-inflammatory properties (42, 43). In addition, LXR activation has been shown to polarise macrophages to the M2 phenotype (44). Mukwaya et al. (45) reported that progressive activation of the LXR/RXR, PPARα/RXRα, and STAT3 pathways after suppression of VEGF signalling could alleviate inflammation and capillary remodelling. Therefore, LXR/RXR activation may also play a protective role in the AR process. In relation to our study, an inflammatory response was clearly observed in GO analysis of DEGs from the AR, ABMR, and TCMR groups (Figures 1B, 2B, 3B), suggesting that the inflammatory response plays an important role in allograft rejection. The upregulation of some other proinflammatory elements was also observed in our analysis, including IL-6 and IL-8 (CXCL-8) signalling pathways (Figure 6). In contrast, the downregulation of some antiinflammatory elements, such as LXR/RXR activation and PPAR signalling, was observed. Thus, we believe that the imbalance of proinflammatory and antiinflammatory elements somehow plays an important role in the promotion of AR.

In addition, we constructed an ROC curve to determine the power of DEGs for the diagnosis of AR (Figures 7C,D). Interestingly, four genes (TNFSF14, TANK, ANKRD33B, and HSPA1B) were identified as effective biomarkers distinguishing AR from non-AR groups. Most of these genes have important biological functions and are closely associated with the immune system and inflammation. TNFSF14 (also called LIGHT) plays an important role in T cell activation and inflammation. It is produced by T cells, which can stimulate T cell proliferation and cytokine production, and is closely associated with T cell-mediated diseases (46, 47). LIGHT-mediated signalling modulates macrophage activity, which may be beneficial for the treatment of chronic inflammatory conditions (48). Wang et al. (49) reported that LIGHT might be a critical cytokine involved in the development of autoimmune inflammatory diseases. HSPA1B (also known as HSP72) has many biological functions. It can enhance STUB1-mediated SMAD3 ubiquitination and degradation and facilitates STUB1-mediated inhibition of TGF-β signalling, which is essential for STUB1-mediated ubiquitination and degradation of FOXP3 in regulatory T cells (Treg) during inflammation (50, 51). Recently, Wang et al. (52) reported that TANK serves as an important negative regulator of NF-κB signalling cascades induced by genotoxic stress and IL-1R/Toll-like receptor stimulation (52). Thus, the potential of these genes as AR biomarkers and their role in AR require further research.

Nevertheless, this study has a few limitations. First, the sample size was relatively small and larger meta studies need to be performed to validate our findings. Second, the transcriptional profiles identified in the current investigation need to be validated using additional RNA-Seq studies of PBMCs and kidney allograft biopsies for validation. Third, patients with high sensitivity must receive immunosuppressive drug treatment before transplantation. Moreover, different patients usually accept different treatment methods and this may have had an unaddressed effect on our results. Future work should take these limitations into account when testing the clinical utility of the identified biomarkers in blinded prospective studies.

Hence, our study showed that several classical pathways and BPs of DEGs, such as complement activation, immune response, and inflammation, were significantly enriched in the AR and non-AR groups. We also found that some pathways and molecules may contribute to the occurrence of AR, whose role in AR has rarely been reported in the past, including the IL-10 signalling pathway, IL-10, CXCL8, and VEGFA. Moreover, we identified a potential 4-gene combination with a ROC AUC of 92.3% (95% CI 82.8–100) for discrimination between AR and non-AR, which requires further validation. Thus, here the characterisation of PBMCs by RNA-Seq and bioinformatics analysis demonstrated the gene signatures and biological pathways associated with patients with AR and non-AR, thereby providing a framework for the discovery of potential biomarkers and an in-depth understanding of acute renal allograft rejection.

## Data Availability Statement

The datasets presented in this study can be found in online repositories. The names of the repository/repositories and accession number(s) can be found below: NCBI with accession number PRJNA782682. (https://www.ncbi.nlm.nih.gov/sra/?term=PRJNA782682).

## Ethics Statement

The studies involving human participants were reviewed and approved by the Institutional Review Board of the Zhejiang University School of Medicine. The patients/participants provided their written informed consent to participate in this study.

## Author Contributions

JC and HJ designed the study and supervised all parts of the study. WX, CW, and HC performed the experiments. WX, LS, and TZ contributed to the collection of samples and clinical data. SH and WX wrote the manuscript. SH, KW, WW, and JQ analysed the data. NS, SR, and HH helped to perform the analysis with constructive discussions. All authors contributed to the article and approved the submitted version.

## Funding

This research was funded by the National Natural Science Foundation of China (grant numbers 81970651 and 81770752) and Sino-German Centre (grant number GZ1572).

## Conflict of Interest

The authors declare that the research was conducted in the absence of any commercial or financial relationships that could be construed as a potential conflict of interest. The reviewer NN declared a shared affiliation with one of the authors, JQ, to the handling editor at time of review.

## Publisher's Note

All claims expressed in this article are solely those of the authors and do not necessarily represent those of their affiliated organizations, or those of the publisher, the editors and the reviewers. Any product that may be evaluated in this article, or claim that may be made by its manufacturer, is not guaranteed or endorsed by the publisher.
